# Caribou balance winter range fidelity and plasticity in response to weather, pregnancy, and summer range conditions

**DOI:** 10.1093/jmammal/gyag039

**Published:** 2026-05-23

**Authors:** Timothy J Fullman, Brian T Person, Shawna Karpovich, Alexander K Prichard, Joelle Hepler, Alain F Zuur

**Affiliations:** The Wilderness Society, 705 Christensen Drive, Anchorage, AK 99501, United States; Department of Wildlife Management, North Slope Borough, P.O. Box 69, Utqiaġvik, AK 99723, United States; Alaska Department of Fish and Game, 1300 College Rd, Fairbanks, AK 99701, United States; ABR, Inc. – Environmental Research & Services, 2842 Goldstream Rd, Fairbanks, AK 99708, United States; Alaska Department of Fish and Game, 1300 College Rd, Fairbanks, AK 99701, United States; Highland Statistics, Ltd, 9 St. Clair Wynd, Newburgh, Aberdeenshire AB41 6FN, United Kingdom

**Keywords:** Arctic, behavior, Caribou, large herbivore, migration, movement, plasticity, *Rangifer tarandus*, site fidelity

## Abstract

In variable environments, animals can remain in place and tolerate changes or move to areas with favorable conditions. Species must balance the costs and benefits of site fidelity and behavioral plasticity in acclimating to rapidly changing environments. We investigated winter fidelity and behavioral plasticity of the Teshekpuk Caribou Herd (TCH) in Alaska. Using GPS telemetry data for 192 female Caribou (*Rangifer tarandus*) from 2004 to 2021, we identified patterns of winter distribution and how they changed compared to reports from 1990 to 2015. We investigated fidelity to wintering areas by comparing observed use of wintering areas with expectations based on area, distance, and sociality. We then examined intrinsic and extrinsic factors correlated with TCH winter distribution patterns using generalized additive mixed models to better understand how resources may influence use of wintering areas for the herd. Caribou used the same 4 wintering areas reported in prior studies, but increased use of the eastern wintering areas recently. TCH Caribou appeared to balance site fidelity and behavioral plasticity. Caribou exhibited some winter fidelity, with reuse of winter areas exceeding expectations, but still often changed wintering areas across years. Distribution among wintering areas varied with both intrinsic and extrinsic factors including summer and fall weather and, to a lesser extent, pregnancy, age, forage condition, and insect harassment. Weather variables had strong effects, with warmer fall air temperatures and lower precipitation corresponding to increased likelihood of animals forgoing migration to the southern mountains and instead overwintering on northern coastal plain tundra. Pregnant Caribou also were more likely to remain on the coastal plain overwinter. The flexibility of Caribou exhibiting both winter fidelity and plasticity may enhance robustness to environmental changes, but increased use of areas near industrial development emphasizes the importance of continued monitoring. Researchers should consider fidelity and behavioral plasticity across scales to inform effective management under changing conditions.

## Teaser

The Teshekpuk Caribou Herd balances winter fidelity and behavioral flexibility. The herd consistently uses 4 wintering areas, but individuals commonly change areas between years. These patterns correspond with weather, pregnancy, and summer range conditions like food availability and insect harassment.

Many animals across the globe face challenges and constraints due to seasonal fluctuations in the environment and use different adaptive strategies to address these obstacles (Varpe 2017; Arnold 2020). From a spatial perspective, animals have 2 options—remain in place and tolerate changing conditions or move to habitats with more favorable conditions. Remaining in place may involve physiological mechanisms such as increased water conservation (Tracy and Walsberg 2001), accumulating body stores of fat and other resources (Blix 2016), or altering body temperature and metabolic performance (Grossman and West 1977; Geiser 2013; Ruf and Geiser 2015; Petit et al. 2017). Tolerance can also be achieved through behavioral adaptations such as shifts in foraging to maximize resource uptake and minimize energetic loss (Nagy 1994), altering timing of reproduction based on resource availability (Lewis and Kappeler 2005; Shine and Brown 2008), or decreasing activity levels to conserve resources (Kenagy 1973; Moen 1976; Joly et al. 2025). In contrast, migratory movements represent an alternative strategy animals use to escape harsh environmental conditions and access additional resources (Alerstam et al. 2003; Dingle and Drake 2007; Avgar et al. 2014).

While migratory behavior is widespread in the animal kingdom, it also encompasses a tremendous diversity of expression (McGuire and Fraser 2014), including classic long-distance round trip movements by land (Holdo et al. 2009; Joly et al. 2019), water (Brown et al. 1995; Lascelles et al. 2014), and air (Egevang et al. 2010; Morten et al. 2025); altitudinal migration across elevation gradients (Albon and Langvatn 1992; Hsiung et al. 2018); vertical migration though aquatic depths (Bandara et al. 2021); and multi-generational migration (Flockhart et al. 2013; Reich et al. 2024). Migratory species often demonstrate fidelity to seasonal ranges or movement routes (Greenwood 1980; Hunter et al. 2003; Horton et al. 2017; Sawyer et al. 2019; Shimada et al. 2020; Morrison et al. 2021). Site fidelity is thought to convey various benefits to animals, such as enhanced fitness due to familiarity with the location of resources, increased access to mates, greater foraging efficiency, or robustness to unpredictable environments (Switzer 1993; Wakefield et al. 2015; Gerber et al. 2019; Shimada et al. 2020; Rebstock et al. 2022). However, site fidelity also has costs and can be maladaptive if environmental or anthropogenic change reduces the quality of sites but strong fidelity hinders use of alternative habitats (Matthiopoulos et al. 2005; Merkle et al. 2022; Le Pla et al. 2024). Indeed, behavioral plasticity can convey benefits for adaptation, individual fitness, and population longevity—especially for species facing rapid environmental change (Parrish 2000; Møller et al. 2008; Beever et al. 2017; Caspi et al. 2022). There is increasing recognition of variable degrees of plasticity in migration and site fidelity behavior (Sandlund and Jonsson 2016; Xu et al. 2021; Verhoeven et al. 2022; Horton et al. 2023). It is thus important to investigate the balance of plasticity and fidelity across individuals and populations to inform conservation and management decisions (Xu et al. 2021), as different management strategies may be needed for species that exhibit a strong fidelity than for those that display a high degree of behavioral plasticity.

Recent work has emphasized the importance of defining expectations in studies of site fidelity to distinguish between behavior consistent with memory-driven fidelity as compared to other, more resource-driven patterns (Picardi et al. 2023). We evaluate patterns of winter distribution and fidelity for barren-ground Caribou (*Rangifer tarandus granti*), which make some of the longest overland migrations in the world (Joly et al. 2019). However, this iconic migratory behavior is combined with a high degree of behavioral plasticity (Xu et al. 2021). Caribou exhibit partial migration (Fullman et al. 2021; Joly et al. 2025), as some individuals migrate while others remain resident year-round. Caribou site fidelity is typically considered to be low (Hinkes et al. 2005; Morrison et al. 2021; Xu et al. 2021), though some herds show regional and seasonal fidelity (Schaefer et al. 2000; Wittmer et al. 2006; Joly et al. 2021), especially during calving (Cameron et al. 2020; Joly et al. 2021).

We first identify patterns of winter distribution for the Teshekpuk Caribou Herd (TCH). This herd exhibits distinctive behavior among Alaskan barren-ground Caribou due to the majority of individuals overwintering on coastal plain tundra rather than migrating south to the Brooks Range mountains and areas beyond (Person et al. 2007; Prichard et al. 2020b; Fullman et al. 2021). We examined the winter distribution of TCH Caribou among 4 wintering areas from 2004 to 2020 and compared this to winter patterns reported in previous studies (1990 to 2005: Person et al. 2007; 2004 to 2015: Fullman et al. 2021). We investigated fidelity to wintering areas by comparing observed use of wintering areas with that expected based on area, distance, and sociality. We hypothesized that observed transition probabilities would exceed those under each alternative mechanism, indicating some degree of fidelity to wintering areas (Fullman et al. 2021), but that fidelity would be moderate, consistent with reports of previous studies (Hinkes et al. 2005; Fullman et al. 2021; Morrison et al. 2021; Xu et al. 2021).

Given our expectation of moderate levels of fidelity, we then examined intrinsic and extrinsic factors correlated with TCH winter distribution patterns to better understand how resources may influence the use of wintering areas for the herd. We hypothesized that Caribou overwintering behavior was influenced by intrinsic factors, including body condition, age, and pregnancy status, as well as extrinsic environmental variation in weather and climate conditions. Migration is energetically costly for individuals (Alerstam et al. 2003; Wikelski et al. 2003; Lennox et al. 2016; Fudickar et al. 2021) and body condition has been shown to influence migratory performance in some species (Duijns et al. 2017) while migratory behavior may help compensate for body condition in other cases (Denryter et al. 2022). Caribou rely on body stores for winter survival, spring migration to calving grounds, and lactation (Fancy 1986; Barboza and Parker 2008; Parker et al. 2009; Taillon et al. 2013). Body condition is influenced by forage conditions and the degree of harassment by biting insects such as mosquitoes (*Culicidae*) and oestrid flies (*Oestridae*), which can alter Caribou behavior, movement patterns, and foraging opportunities, with consequences for reproduction and survival (Joly et al. 2020; Johnson et al. 2021, 2022). Pregnancy increases energetic costs for Caribou (Barboza and Parker 2008), is associated with higher cortisol levels in late winter (Joly et al. 2015), and may influence risk-taking behavior such as avoidance of areas with greater predator levels (Barten et al. 2001). The probability of pregnancy in Caribou varies with age (Thomas and Barry 1990; Adams and Dale 1998). Age may also affect migration propensity, as is seen in some ungulates (Singh et al. 2012).

In addition to body condition, pregnancy, and age, external environmental factors also may affect Caribou behavior. Fall migration propensity in the nearby Western Arctic Herd is influenced by air temperature and snowfall (Cameron et al. 2021). Such behavioral decisions regarding initiation or cessation of migration will strongly influence subsequent winter use. Indeed, winter distribution in the neighboring Central Arctic Herd is also influenced by snow levels (Pedersen et al. 2021). Patterns in such environmental conditions may also be influenced by broader climate conditions. For example, the Arctic Oscillation climate index is related to shifting surface pressures, with the jet stream remaining farther north during positive phases and further south during negative phases, leading to altered winter temperatures (Higgins et al. 2000; Higgins et al. 2002) and precipitation (Simpson et al. 2002). High values of this index correlate with reduced snow in the western Arctic (Gurarie et al. 2019) and with negative population growth for Svalbard Reindeer (Aanes et al. 2002).

We developed 12 non-exclusive hypotheses for factors that may influence winter distribution of the TCH (Table 1). These include the influence of body condition, pregnancy status, age, weather, and climate conditions, as well as cumulative effects across intrinsic and extrinsic factors. We tested among these hypotheses using generalized additive models applied to telemetry data for the TCH. We ran these models at 2 scales. First, Caribou of the TCH may remain on the arctic coastal plain tundra all winter or may migrate south to the Brooks Range mountains. These offer tradeoffs of resources and risks. While the mountains offer higher lichen densities, they also require longer migrations, have deeper snow, and higher predator densities (Carroll 2015; Poley et al. 2018; Klimstra 2020; Macander et al. 2022). In contrast, the coastal plain has low predator densities but also lower lichen cover with shallower, but windblown and dense, snow conditions. It can also experience severe winter weather, which may result in large mortality events in extreme cases (Dau 2005; Bieniek et al. 2018). Second, prior studies of TCH distribution have noted 4 wintering areas used by the herd (Person et al. 2007; Fullman et al. 2021). Even among caribou that remain overwinter on coastal plain tundra, some make shorter migrations to other parts of the coastal plain (Fullman et al. 2021). At this finer scale, we examined how our hypotheses explained observed use of the different wintering areas. Together, these investigations provide an opportunity to better understand spatial fidelity and winter distribution patterns in the TCH and what intrinsic and extrinsic factors are correlated with these behaviors. Understanding the characteristics, mechanisms, and alterations in migratory behavior and space use can help suggest strategies to support management and conservation efforts (Xu et al. 2021).

**Table 1:** *A priori* models defining hypotheses of factors that may influence winter distribution of Caribou.

Model	Expression	Hypothesis
**M1**	NDVI + Insect	Body stores (combination of forage availability and energetic costs of insect harassment) affect Caribou overwintering behavior
**M2**	Age	Older individuals accumulate increased body stores and have greater experience to facilitate migration to overwinter sites
**M3**	Pregnancy	Pregnancy exerts large energetic demands and may alter risk tolerance
**M4**	Pregnancy + Age + Pregnancy × Age	Pregnancy probability varies with age
**M5**	NDVI + Insect + Pregnancy + Age + Pregnancy × Age	Body stores and demands are cumulative
**M6a**	sTemp + sPrecip + fTemp + fPrecip	Harsh weather (before or during migration) affects the likelihood of initiating migration or of abandoning an attempt to leave the coastal plain
**M6b**	fTemp + fPrecip	Only fall weather matters
**M6c**	sTemp + sPrecip	Only summer weather matters
**M7**	NDVI + Insect + Pregnancy + Age + Pregnancy × Age + sTemp + sPrecip + fTemp + fPrecip	Weather effects are moderated by body condition and energetic demands
**M8**	sAO + fAO	Broad-scale climate conditions affect overwintering behavior
**M9**	NDVI + Insect + Pregnancy + Age + Pregnancy × Age + sAO + fAO	Body condition and energetic demands vary with broad climate factors
**M10**	Null	None of the covariates affect overwintering behavior

We evaluated models at a coarse scale comparing Caribou that migrate south to the Brooks Range mountains with those remaining overwinter on coastal plain tundra and at a fine scale comparing use of specific wintering areas within the coastal plain and Brooks Range. Expressions indicate the dependent variable portion of the regression equations used to evaluate Caribou overwintering behavior. Lower case letters preceding covariates indicate seasonal values (s, summer; f, fall). AO, Arctic Oscillation climate index; Insect, insect harassment index; NDVI, summer normalized difference vegetation index; Precip, precipitation; Pregnancy × Age, interaction between pregnancy status and age; Temp, air temperature.

## Methods

### Study area and species

**Fig. 1:**
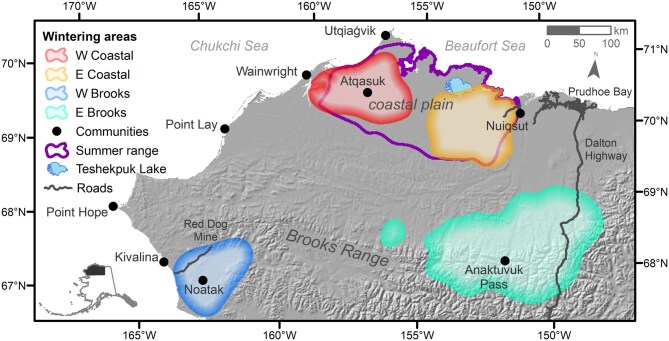
Study area depicting the 4 wintering areas used by Teshekpuk Caribou Herd females in northern Alaska, 2004-2020.

The TCH is moderately sized compared to other Alaskan herds, with 61,600 animals reported in 2022 (ADFG 2025). The herd derives its name from its primary calving area around Teshekpuk Lake, near the coast of the Beaufort Sea (Fig. 1). Calving occurs in late May and early June, mostly around Teshekpuk Lake with some calving further west (Carroll et al. 2005; Person et al. 2007; Wilson et al. 2012; Prichard et al. 2019). The TCH remains on the coastal plain throughout the summer, aggregating near the coast to seek relief from biting insects before spreading out across the coastal plain to forage after insect harassment abates in late summer (Fig. 1; Person et al. 2007; Wilson et al. 2012). The herd splits in the fall, with some animals remaining resident around Teshekpuk Lake, others making short migratory movements within the coastal plain, and some Caribou traveling hundreds of kilometers south to leave the coastal plain and overwinter in the Brooks Range mountains and beyond (Person et al. 2007; Fullman et al. 2021).

Caribou of the TCH use 4 primary wintering areas (Fig. 1; Person et al. 2007; Fullman et al. 2021), which span a range of environmental conditions (Table 2; Supplementary Data SD1). These wintering areas stretch from sea level on the coastal plain to over 2,000 m elevation in the Brooks range. All 4 areas have relatively similar ranges of winter air temperature but vary in snow depth and wind speed, with deeper snow and lower windspeed in the Brooks Range compared to the coastal plain (Table 2; Supplementary Data SD1). Precipitation levels are generally similar, except for higher precipitation in West Brooks. Petroleum exploration and production are expanding into areas used by the TCH during summer and winter (Person et al. 2007; Wilson et al. 2012; Fullman et al. 2021), increasing the urgency of understanding habitat use and movement patterns to inform management decisions.

**Table 2:** Mean (min, max) environmental characteristics of the 4 primary wintering areas for the Teshekpuk Caribou Herd.

	W Coastal			E Coastal			W Brooks			E Brooks		
**Area (km^2^)**	12,423			14,234			10,027			36,218		
**Elevation (m)**	21	(–6,	79)	41	(–4,	172)	160	(2,	1131)	931	(234,	2441)
**Air temperature (°C)**	–20.4	(–23.8,	–16.6)	–21.3	(–24.8,	–17.3)	–18.0	(–22.3,	–13.5)	–20.4	(–23.8,	–17.1)
**Precipitation (mm)**	21.5	(14.2,	28.9)	20.2	(13.5,	24.8)	43.8	(24.5,	64.2)	26.0	(17.3,	35.4)
**Snow depth (m)**	0.4	(0.3,	0.5)	0.4	(0.3,	0.6)	0.6	(0.4,	0.9)	0.7	(0.4,	0.9)
**Wind speed (m/s)**	5.4	(4.6,	6.1)	4.8	(4.2,	5.5)	4.0	(3.5,	4.6)	2.0	(1.7,	2.2)
**Lichen cover (%)**	1.5	(0.0,	19.3)	2.0	(0.0,	19.6)	2.8	(0.0,	31.8)	4.2	(0.0,	45.2)

Elevation data comprise 32 m elevation pixels from the ArcticDEM mosaic dataset (Porter et al. 2023). Weather data represent the winter season (11 November to 1 May) mean values for each analysis-year from 2004 to 2020 from the hourly ERA5-Land reanalysis dataset (Muñoz-Sabater et al. 2021). Lichen data summarize 30 m pixels of modeled lichen top cover for 2020 (Macander and Nelson 2022). See Supplementary Data SD1 for distributions of each variable.

### Data collection and preparation

Caribou were captured using a net gun from an R44 helicopter in late June and early July between 2004 and 2020. Captured Caribou were fitted with GPS collars (various TGW- models; Telonics, Mesa, Arizona) and were recaptured as needed based on the battery life of the collar. All captures were conducted under Alaska Department of Fish and Game Institutional Animal Care and Use Approval #2007-13 and subsequent renewals and align with American Society of Mammalogist guidelines (Sikes et al. 2016). Location data spanned 2004 to 2021; however, we excluded July 2005 to June 2006 due to all individuals having their GPS collars replaced with lower fix rate and accuracy Platform Terminal Transmitter (PTT) collars (Fullman et al. 2021).

We filtered the telemetry data to remove locations that were duplicated, post-mortality, or presumed erroneous based on the combination of distance, rate, and angle (Prichard et al. 2014). We then divided data for each Caribou into “analysis-years” stretching from 1 July of a year to 30 June of the subsequent year (Fullman et al. 2021). These were named by their start year, such that the 2019 analysis-year spanned 1 Jul 2019 through 30 Jun 2020. As with Fullman et al. (2021), we excluded analysis-years with a duration less than 290 d (Cagnacci et al. 2016) and those with gaps in location information greater than 2 consecutive weeks. Collars recorded Caribou locations at varying pre-programed fix intervals ranging from every 2 to 12 h. We standardized all collars to a 2-fix-per-day (approximately 12-h) frequency. Caribou occasionally switched herds temporarily or for extended periods, adopting movement and calving behavior of a different herd (Prichard et al. 2020b). We removed 16 such analysis-years that did not reflect movement patterns for the TCH.

### Winter distribution

**Fig. 2:**
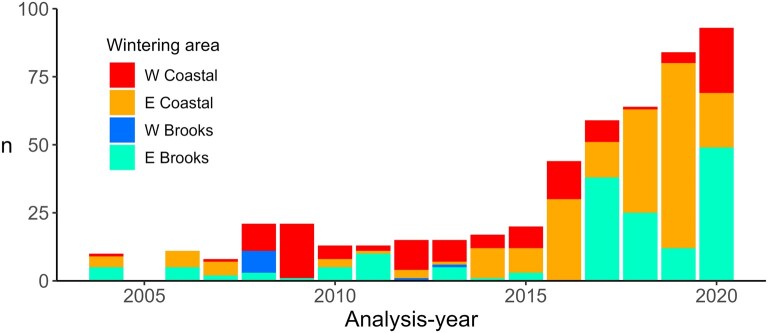
Wintering area distribution over time by Teshekpuk Caribou Herd females in northern Alaska, analysis-years 2004-2020. Analysis-years span 1 July of the indicated year through 30 June of the following year.

To identify candidate wintering areas, we created a population-level winter utilization distribution (UD) for the TCH. This procedure used only winter locations, identified using individual-specific winter start and end dates for migrants based on first passage time—net squared displacement analysis (Fullman et al. 2021). In brief, the first passage time—net squared displacement approach combined tortuosity of movement with changes in movement areas to subdivide an annual movement track into seasonal movement behaviors and distinguish migratory, resident, and other movement strategies (Le Corre et al. 2014, 2017; Fullman et al. 2021). We used the median winter start and end dates of migrants (11 November and 1 May, respectively) to identify winter locations for residents and other non-migratory movement behaviors where start and end dates could not be identified based on movement. The winter locations were then used to calculate annual UDs using kernel density estimation (KDE) in the “adehabitatHR” R package (version 0.4.19; Calenge 2021), selecting the optimal bandwidth for the KDE following Kie’s (2013)  *ad hoc* approach (Fullman et al. 2021). Because sample size varied widely over time (Fig. 2), we standardized each annual winter UD to sum to 1 and then averaged the results to yield an overall population-level winter UD for the full 2004 to 2020 period. This approach helped prevent the higher sample sizes in the later period from biasing the population-level distribution toward those areas, better reflecting herd-level distribution over time.

We identified 4 wintering areas used by the TCH, as reported in previous studies (Person et al. 2007; Fullman et al. 2021). Following Fullman et al. (2021), we distinguished 2 wintering areas in the highly used coastal plain based on 50% UD contours (W Coastal and E Coastal; Fig. 1). In the more lightly used Brooks Range wintering areas we used wider contours, with an 85% UD contour used to distinguish the East Brooks (E Brooks) wintering area and a 95% contour distinguishing the sparsely used West Brooks (W Brooks) wintering area (Fig. 1). Caribou in each analysis-year were assigned to the wintering area with which their individual winter UD had the greatest overlap (Fullman et al. 2021).

Our coarse-scale examination of factors associated with winter distribution involved comparing Caribou that remained on the coastal plain overwinter with those that migrated south to the Brooks Range mountains. This comparison necessitated distinguishing animals remaining on the coastal plain from those that migrated south. While Caribou reduce movement rates during the winter (Prichard et al. 2014; Joly et al. 2020, 2025), a small number of individuals (*n *= 9, 1.8% of all analysis-years) moved mid-winter between 2 wintering areas. To ensure that the behavior of animals that migrated south was adequately reflected, we visually reviewed the UDs for these individuals and assigned them to the non-summer wintering area as long as they spent at least 1 month outside of E Coastal.

### Winter fidelity

Site fidelity reflects a return to previously used locations (Switzer 1993). We calculated transition probabilities using the “TraMineR” R package (version 2.2-0.1; Gabadinho et al. 2011) for all Caribou with multiple analysis-years of data to indicate the likelihood of Caribou returning to a given wintering area versus transitioning between wintering areas in subsequent years (Fullman et al. 2021). We compared observed transition probabilities against those generated under 4 null models (Picardi et al. 2023) reflecting different mechanisms that could explain Caribou winter distribution patterns in the absence of site fidelity (Table 3). Our “equal” null model reflected the possibility that each wintering area received an equal probability of use (Table 3). We derived the “area” and “distance” null models from the theory of island biogeography (MacArthur and Wilson 1963, 1967). The “area” null model posited that the probability of use of each wintering area was equal to its proportion of the total winter contour area, such that larger wintering areas had a higher probability of use, analogous to more species arriving on larger islands due to chance (Table 3). The “distance” null model reflected expectations of more species arriving on closer islands due to chance, with the probability of use of each wintering area inversely proportional to the distance between its KDE-weighted centroid and that of the 95% UD contour for the population-level TCH summer range (which we calculated following the same approach as the winter range; Fig. 1). The final null model, “population,” reflected social processes in which Caribou, as gregarious animals, were attracted to conspecifics (Hendrix et al. 2024). In this model, the probability of use of each wintering area was equivalent to its proportional use across all winters (Table 3).

**Table 3:** Null model expectations for probability of use of 4 wintering areas in northwestern Alaska by Teshekpuk Caribou Herd females, 2004 to 2020.

Null model	W Coastal	E Coastal	W Brooks	E Brooks
**Equal**	0.25	0.25	0.25	0.25
**Area**	0.17	0.20	0.14	0.50
**Distance**	0.29	0.31	0.17	0.24
**Population**	0.24	0.42	0.02	0.32

We generated our null model expectations by taking each Caribou with multiple years of recorded winter distribution and assigning a wintering area to each analysis-year for which an observation had been made, using a random-weighted assignment based on the null model being tested. We calculated the transition probabilities between each wintering area using these simulated data, just as had been done with the observed data. We repeated this process 500 times per null model and calculated the mean and 95% confidence interval of transition probabilities. Observed transition probabilities that did not overlap the confidence intervals of a given null model were considered significantly different from the expectation under that null model. We also fed the observed counts in each wintering area and the null model probabilities into a multinomial distribution using the “dmultinom” function in R (version 4.2.2; R Core Team 2024) to calculate the probability that the observed data would be yielded by chance under each null model.

### Factors associated with winter distribution

We investigated how intrinsic and extrinsic factors influence Caribou use of wintering areas at 2 scales: (i) whether a Caribou remained on the coastal plain during the winter or migrated south; and (ii) use of the 4 distinct wintering areas. Preliminary analyses revealed non-linear patterns, so we used a generalized additive mixed modeling (GAMM) approach with smoothers fit for non-linear responses to the covariates (Wood 2017). Smoothers, in the form of cubic regression splines, were used for all model covariates other than pregnancy status, which we incorporated as a standard parametric variable based on preliminary analysis. Probability of migrating south to the mountains was modeled with a binary response variable. Probability of use of the different wintering areas was modeled with a multinomial model, using E Coastal as the reference class to represent individuals that remained in the general vicinity of the calving and summer range during the winter. Animals rarely overwintered in the W Brooks area (*n *= 10), leading us to drop these observations from the multinomial analysis to avoid unstable estimates. A random intercept for the individual was included in both models to account for observations in multiple analysis-years from the same Caribou. Models were run using the “mgcv” package in R (version 1.9-1, Wood 2011, 2017).

Intrinsic factors included pregnancy status and age. We inferred pregnancy status during the winter being considered based on whether a Caribou had a calf in the spring at the end of that analysis-year. Trained Alaska Department of Fish and Game staff flew aerial surveys during the peak calving season (30 May to 11 June) to determine whether collared females had produced a calf or were likely to do so. This determination was based on observations of calf at heel, retention of antlers, or distended udders—metrics that have been demonstrated to be highly accurate for Caribou parturition (Whitten 1995; Johnson et al. 2022). We recognize that this approach may misclassify some Caribou if their pregnancy was terminated during the winter. Age values represented the minimum age for each individual. Known ages were used where available for individuals first captured as yearlings or those with dental records to allow aging. For all other individuals, we estimated a minimum age at capture of 3 yr for Caribou collared as adults. We also included the interaction of age with pregnancy status to account for pregnancy likelihood varying with age (Thomas and Barry 1990; Adams and Dale 1998).

Extrinsic factors consisted of air temperature, precipitation, insect harassment, vegetation greenness, and climate. Air temperature and precipitation data were obtained from the hourly ERA5-Land reanalysis dataset (Muñoz-Sabater et al. 2021), accessed from Google Earth Engine (Gorelick et al. 2017; Muñoz-Sabater 2019) using the “rgee” R package (version 1.1.5; Aybar et al. 2020, Aybar 2022). Temperature and precipitation data were sampled at Caribou locations and timesteps and combined into seasonal averages for each Caribou (Gurarie et al. 2024), using median season dates for migrants (summer, 1 July to 1 October; fall, 2 October to 10 November). We derived an insect harassment index reflecting the proportion of observations during the summer season in which biting insects were considered active based on equations relating air temperature and wind speed to mosquito and oestrid fly activity (Russell et al. 1993), with values above 0.5 considered active (Wilson et al. 2012). Wind speed data used in calculating the harassment index were also from ERA5 and were matched with the corresponding air temperature records at each Caribou location. Vegetation was represented by MCD43A4 MODIS-derived Normalized Difference Vegetation Index (NDVI) data (Schaaf and Wang 2015), obtained from Google Earth Engine using “rgee.” As with the air temperature and precipitation data, NDVI values were obtained at Caribou locations and converted to a summer seasonal average, with values <0.1 set to NA prior to averaging to avoid impacts of snow, ice, or bare areas (Weier and Herring 2000; Pedersen et al. 2018). Finally, we obtained monthly Arctic Oscillation (AO) climate index data from the National Weather Service Climate Prediction Center (NOAA/NWS 2005), which we averaged to seasonal means. Derived data used in our analyses are available via the Dryad repository (Fullman et al. 2026).

Our model evaluation approach proposed *a priori* hypotheses to explain the response variable of interest, examining support among these using a web of evidence approach, which compared the competing hypotheses using multiple model selection approaches and diagnostics and then identified influential covariates based on effective degrees of freedom and overlap of 95% simultaneous confidence intervals with zero. We evaluated 12 models representing biologically based hypotheses explaining Caribou overwintering behavior (Table 1), which we applied at both coarse and fine scales of analysis. At both scales, models were compared based on Akaike’s Information Criterion (AIC), deviance explained, and k-fold cross validation with 5 folds, calculating Area Under the Receiver Operating Characteristic Curve (AUC), log-loss, percent correctly classified, sensitivity, and specificity on each fold. Deviance explained is somewhat analogous to *R*^2^ values of generalized linear models, indicating how well a model fits the data compared to that of the full model, with higher values indicating better model performance (Zuur et al. 2009). A model’s ability to distinguish between outcomes is indicated by its AUC score, with higher values, closer to the maximum of 1, indicating better model performance (Hanley and McNeil 1982; Janssens and Martens 2020). Log-loss reports the accuracy of probabilistic predictions, with lower values indicating better performance (Aggarwal et al. 2021). Percent correctly classified indicates the percentage in which the modeled and observed response values match, with higher values indicating better model accuracy (Freeman and Moisen 2008). Sensitivity and specificity reflect the true positive and negative rates, respectively, with higher values indicating better model performance (Freeman and Moisen 2008). We simulated random effects for unseen individuals (i.e., those occurring in test but not training data for a particular cross-validation fold) from a normal distribution with a mean of zero and variance equal to the estimated random effect variance. There is no single agreed-upon statistical approach for best evaluating models, with critiques raised about each approach (Bradley 1997; McCullagh and Nelder 1998; Guthery et al. 2005; Lobo et al. 2008). We thus sought consistency across various model validation methods to identify a set of top-ranked candidate models that we evaluated for influential covariates.

Upon identifying top-ranked candidate models, we examined each covariate’s influence based on multiple metrics. The effective degrees of freedom (edf) of each covariate with a regression spline provided an indicator of the importance and degree of non-linearity of the estimated effects. Values close to zero were not considered influential in the model, indicating a lack of effect of the corresponding covariate, while covariates with edf values around 1 had a relatively linear effect on the response variable, and those with higher values had non-linear effects (Zuur et al. 2009). We also examined partial effect plots for each smoother using simultaneous 95% confidence intervals (Sison and Glaz 1995; Agresti et al. 2008). Variables whose confidence intervals did not overlap zero were taken to have an important effect on the response variable. We then plotted the effect of each important variable at the response scale while holding all other variables at their mean values. We applied model validation to assess whether the candidate models complied with their underlying assumptions. This included use of the “DHARMa” R package (version 0.4.6, Hartig 2022) to obtain (simulation-based) scaled quantile residuals, which we checked for non-linear patterns and homogeneity.

## Results

The telemetry dataset contained locations for 192 female TCH Caribou. Individual Caribou had between 1 and 9 analysis-years of data (mean = 2.6, median = 2.0), resulting in a total of 508 analysis-years across all Caribou from 1 July 2004 to 30 June 2021. The number of animals varied over time from 8 to 93 Caribou collared per year (mean = 31.8, median = 18.5; Fig. 2), except for 2005, which was omitted as noted above.

While there was substantial variability in the winter UDs from year to year (Supplementary Data SD2), the overall population-level winter UD representing 2004 to 2020 showed continued use of the same general 4 areas reported previously for the TCH (Person et al. 2007; Fullman et al. 2021; Supplementary Data SD3). Winter contours for 2004 to 2020 showed 82% to 100% overlap with those identified from 2004 to 2015 (Supplementary Data SD4). The W Coastal contour was smaller in 2004 to 2020, while all other contours were larger than in 2004 to 2015 (Supplementary Data SD4 to SD5). Where winter assignments overlapped between this study and Fullman et al. (2021; i.e., analysis-years 2004 to 2015), assignments were identical, indicating that use of the updated winter contours did not lead to divergent wintering area classification.

Multiple wintering areas were used each year by collared TCH Caribou (Fig. 2). Overall, the E Coastal area was used most often, followed by E Brooks and W Coastal (Supplementary Data SD6). This is a marked shift from patterns between 2004 to 2015 (Fullman et al. 2021) and reflected increased use of the E Coastal and E Brooks wintering areas in the 2016 to 2020 analysis-years (Supplementary Data SD6). The W Brooks area was rarely used, not showing any use by collared TCH Caribou from 2016 to 2020 (Fig. 2; Supplementary Data SD6).

### Winter fidelity

**Fig. 3:**
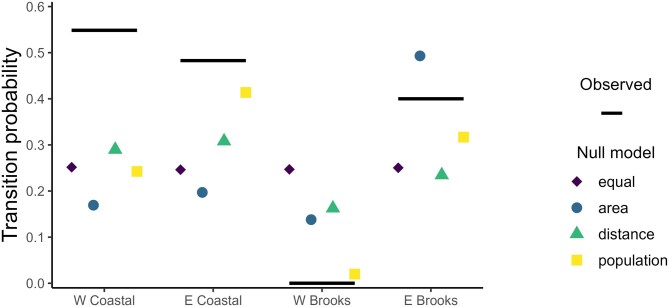
Observed (lines) and expected (dots) transition probabilities for reuse of the 4 wintering areas by Teshekpuk Caribou Herd females in northern Alaska, 2004 to 2020. Observed probabilities of wintering area reuse were compared to expectations under 4 null models: equal probability of use, area-weighted probability of use, distance-weighted probability of use, and population-weighted probability of use. Confidence intervals are included as colored lines above and below each null model mean value, but are too small to see clearly (see Supplementary Data SD7 for these values). While only probabilities of reusing the same wintering area in subsequent winters are depicted here, observed transition probabilities for switching wintering areas also diverged from null expectations in almost every instance (Supplementary Data SD7).

Observed transition probabilities for reuse of wintering areas diverged from expectations under each null model (Fig. 3; Supplementary Data SD7), in alignment with our hypothesis of some degree of fidelity. Observed probabilities of reuse of the W Coastal and E Coastal wintering areas were higher than expected under all null models while observed probabilities of reuse were lower than expected for W Brooks (Fig. 3). For E Brooks, most null models predicted a lower probability of reuse than was observed, but the area-based model predicted higher reuse than shown by collared Caribou (Fig. 3). Comparing observed patterns of winter use with null model expectations under the multinomial distribution indicated very low probabilities of obtaining the observed data under the null models (Supplementary Data SD8). The exception to this was the first few years of recorded data when sample sizes were lower, but even here, all probabilities were less than 0.15 (Supplementary Data SD8). It was very unlikely that the use of the 4 wintering areas by collared Caribou occurred by chance under our null models. Nonetheless, observed transition probabilities for re-use of winter areas were <0.6 for all wintering areas, aligning with our hypothesis of moderate fidelity.

### Winter distribution factors

A total of 393 analysis-years for 167 Caribou were included in the analysis of coarse-scale factors associated with winter behavior, reflecting analysis-years for which all covariate data were available. Parturition surveys were not conducted in 2007, and not all individuals could be relocated during each survey, which reduced the sample size. Of the records included in the coarse analysis, 253 (64.4%) were for pregnant animals. The sample size was further reduced in the fine-scale wintering area analysis due to the exclusion of W Brooks as a consequence of low sample size. The fine-scale analysis included the same 167 Caribou with 384 analysis-years, of which 249 (64.8%) were for pregnant animals.

**Fig. 4:**
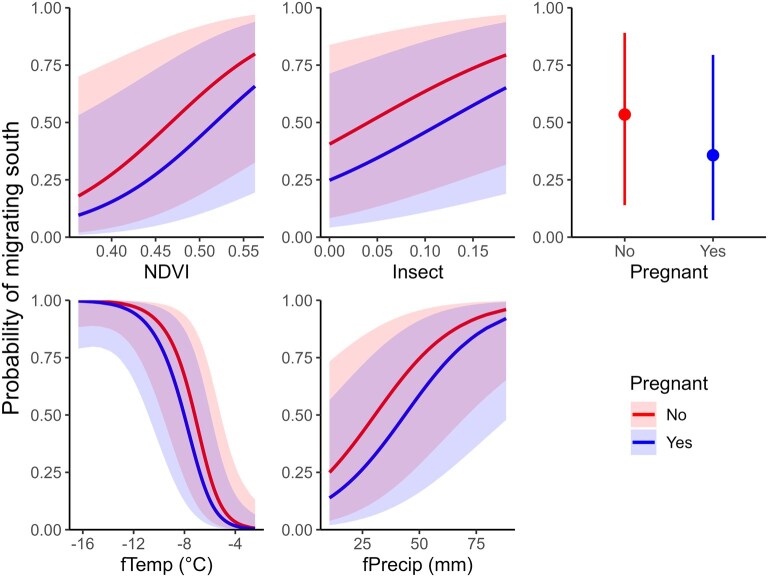
Relationship between important covariates and the probability of Teshekpuk Caribou Herd females leaving the coastal plain over winter in northern Alaska, 2004 to 2020. Effects are depicted holding all other variables at their means, with results shown by pregnancy status. Lower case letters preceding covariates indicate seasonal values (s, summer; f, fall). Insect, insect harassment index; NDVI, summer normalized difference vegetation index; Temp, air temperature; Precip, precipitation.

Model validation results and “DHARMa” residuals indicated no significant deviations from model assumptions at either scale. Summary statistics for coarse-scale models of the probability of a Caribou migrating south to the Brooks Range overwinter indicated support for a single candidate model, M7 (Supplementary Data SD9). Regression spline edf values (Supplementary Data SD10) and partial effect plots (Supplementary Data SD11) indicated important effects of summer NDVI, insect harassment, fall temperature, and fall precipitation. The probability of a Caribou migrating to the mountains increased with greater exposure to summer NDVI, insect harassment, and fall precipitation and decreased with warmer fall air temperature (Fig. 4). Pregnancy status also had a 95% confidence interval that did not overlap zero (Supplementary Data SD10). Non-pregnant Caribou showed increased probabilities of migrating to the mountains, though the response showed a high degree of overlap with pregnant individuals (Fig. 4).

**Fig. 5:**
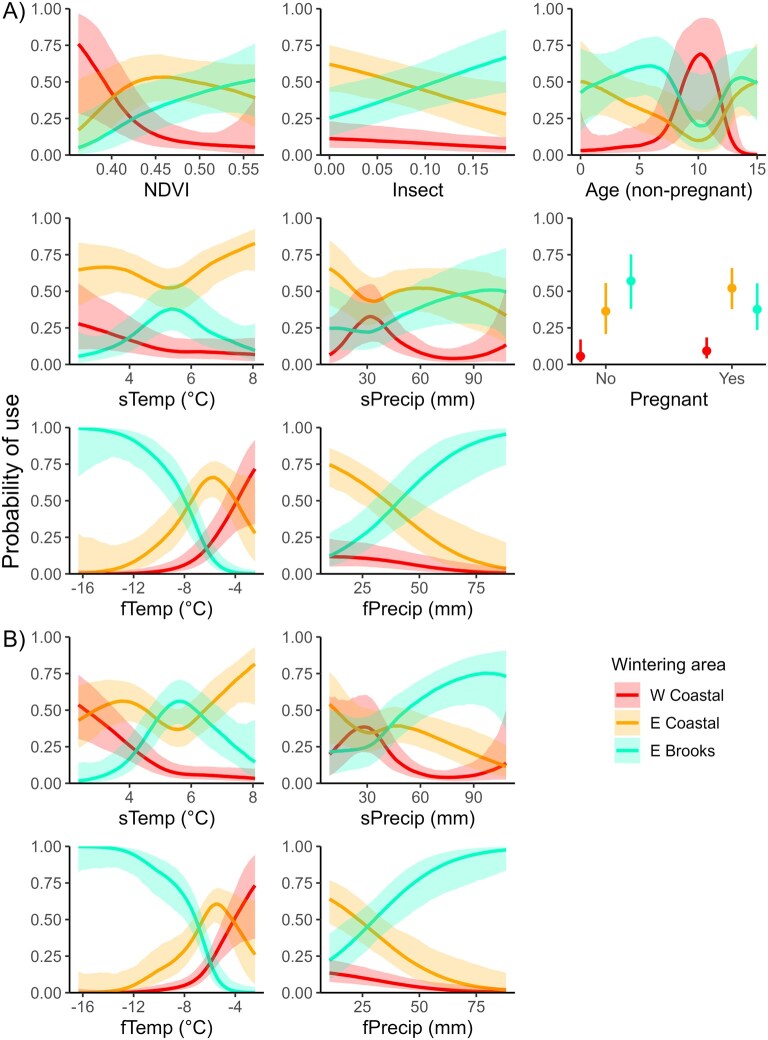
Relationships of important covariates under A) M7 and B) M6a with the probability of overwintering in W Coastal (red), E Coastal (orange), and E Brooks (turquoise) by Teshekpuk Caribou Herd females in northern Alaska, analysis-years 2004 to 2020. W Brooks was not included in the models due to low sample size. See Table 1 for model definitions. Effects are depicted holding all other variables at their means. Lower case letters preceding covariates indicate seasonal values (s, summer; f, fall). Insect, insect harassment index; NDVI, summer normalized difference vegetation index; Precip, ­precipitation; Temp, air temperature.

For the fine-scale analysis of using specific wintering areas, model comparison indicated support for 2 competing models, M7 and M6a (Supplementary Data SD12). Weather variables occurred in both models and exhibited similar responses across models (Supplementary Data SD13 and SD14). Warmer summer air temperatures were associated with decreased likelihood of Caribou overwintering in W Coastal, increased overwintering in E Brooks at moderate temperatures, and increased use of E Coastal at the warmest temperatures (Fig. 5). Greater summer precipitation corresponded to lower probabilities of overwintering in W Coastal, while the likelihood of using E Brooks increased. Colder fall air temperatures were associated with a higher likelihood of overwintering in E Brooks, while use of E Coastal was maximized at more moderate-warm temperatures and W Coastal at the warmest temperatures. W Coastal overwintering showed little response to fall precipitation, while E Coastal and E Brooks showed inverse relationships, with increased probability of overwintering in E Coastal at lower fall precipitation values and in E Brooks at higher precipitation. Non-weather covariates were only included in M7, suggesting that while they may explain some of the observed patterns, their influence is likely not as strong as that of the weather variables. Exposure to greater NDVI values corresponded to lower likelihood of a Caribou overwintering in W Coastal and increased the likelihood of overwintering in E Coastal or E Brooks, with likelihood of overwintering in E Coastal peaking at moderate NDVI values (Fig. 5). Greater insect harassment aligned with higher likelihood of overwintering in E Brooks compared to E Coastal but had little relationship with the probability of using W Coastal (Fig. 5). Age only had an important effect on non-pregnant individuals, with probability of overwintering in W Coastal increasing sharply for non-pregnant animals around 10 yr of age and concurrent decreases in likelihood of use of E Coastal and E Brooks (Fig. 5). Finally, pregnant animals were less likely to use E Brooks overwinter than to remain in E Coastal, but use of W Coastal was not affected (Fig. 5; Supplementary Data SD13).

## Discussion

Behavioral plasticity can help species adapt to rapidly changing environments (Xu et al. 2021; Merkle et al. 2022). Our investigation of winter fidelity in female Caribou of the TCH affirms the plasticity of individual migration decisions previously reported for the herd (Fullman et al. 2021), showing that individuals vary their use of wintering areas from year to year, corresponding to weather conditions and other factors. Nonetheless, our analyses also revealed that reuse patterns exceed null model predictions, indicating that some degree of winter fidelity is maintained for the TCH. In addition to identifying prevalence and potential mechanisms of winter distribution, it is important that researchers consider fidelity and behavioral plasticity across various spatial scales to inform effective management under changing conditions.

### Winter distribution factors

We found that Caribou winter distribution is associated with multiple extrinsic and intrinsic factors. Models indicated influences of weather variables, NDVI, insect harassment, and pregnancy status at both a broad scale of remaining on the coastal plain versus migrating south to the mountains and at finer scales of using specific wintering areas, suggesting possible mechanisms of TCH winter distribution that warrant further investigation. We found a strong relationship between weather variables and winter distribution of Caribou, aligning with patterns observed in other ungulate species. Across 27 ungulate species, interannual variation in climate and weather patterns was among the most frequent correlates of migration change (Xu et al. 2021). In our study, air temperature and precipitation—especially during the fall season—were both associated with winter behavior of the TCH. Warmer fall air temperatures corresponded to a reduced probability of migrating to the mountains overwinter at a coarse scale (Fig. 4) and increased probabilities of Caribou using the 2 coastal plain wintering areas at a fine scale (Fig. 5). Similarly, warmer summer air temperatures were associated with higher use of the E Coastal wintering area (Fig. 5). A study of the neighboring Western Arctic Herd found that warmer fall air temperatures led to reduced migratory movement when snow was absent or shallow (Cameron et al. 2021). While the study did not specifically look at winter behavior, such responses could lead to similar patterns to those we observed. The TCH range has seen substantial warming of average annual near-surface air temperature between 1997 and 2020 (Watts et al. 2025), which may help explain recent observations of increased use of the E Coastal wintering area. If such patterns continue, it could have implications for Alaska Native peoples that rely on Caribou for cultural connections, social well-being, and food security (Borish et al. 2022). For example, the community of Anaktuvuk Pass lies within the middle of the E Brooks wintering area (Fig. 1) and is heavily dependent on Caribou, which comprise a large proportion of their annual subsistence harvest (Bacon et al. 2009; Martin 2015; Berman et al. 2021). If recent increases in air temperature continue and Caribou shift their winter distribution in response, this could reduce food security for Anaktuvuk Pass while potentially increasing the availability of Caribou for more northern communities like Nuiqsut and Utqiaġvik.

Greater fall precipitation corresponded to a higher probability of Caribou leaving the coastal plain overwinter to migrate south to the mountains (Fig. 4), which primarily reflected a switch in fine-scale distribution from E Coastal to E Brooks (Fig. 5). Fall migratory movements in the Western Arctic Herd can be triggered by early snowfall, though as snow accumulates more deeply movement propensity of Caribou declines (Cameron et al. 2021). A greater likelihood of Caribou moving to the E Brooks under conditions of greater fall precipitation may suggest that TCH Caribou follow a similar pattern. While precipitation has increased in Alaska over more than half a century (Wendler et al. 2017), there are some indications that the balance between snow and rain may be shifting (McAfee et al. 2014; Littell et al. 2018). Incidents of rain-on-snow are increasing across the Arctic and Alaska (Bieniek et al. 2018; Pan et al. 2018; Du et al. 2025), which can hinder the ability of Caribou to access winter forage (Hansen et al. 2011, 2019; Forbes et al. 2016) and reduce spring body mass, which has consequences for reproduction and survival (Albon et al. 2017). A large rain-on-snow event in the winter of 2003 led about a third of the TCH to overwinter in the Arctic National Wildlife Refuge, over 400 km east of their typical winter ranges (Carroll 2005; Person et al. 2007), which was accompanied by high mortality (Carroll 2005). It will thus be important to conduct further mechanistic investigation into how changes in the precipitation regime affect Caribou winter distribution and other behaviors.

Summer vegetation productivity and insect harassment both appear to influence TCH winter distribution. These relationships may reflect the roles of these extrinsic factors on body condition and the relative costs and benefits of migration. Arctic Caribou alter their selection for different forage components as vegetation conditions change throughout the summer and in response to insect activity (Johnson et al. 2021). Both forage and insects, in turn, affect survival and reproduction of Caribou (Johnson et al. 2022) and, as our study suggests, winter distribution. Increasing exposure to NDVI values in summer aligned with a greater probability of migrating south to the Brooks Range mountains overwinter (Fig. 4). There was also a fine-scale shift from a greater likelihood of overwintering in the W Coastal wintering area at low NDVI values, to E Brooks and E Coastal areas at higher values (Fig. 5). These patterns may reflect exposure to increased vegetation productivity, allowing accumulation of additional body stores to fuel longer-distance migration. Caution should be taken, however, in going too far with this interpretation, as NDVI does not map linearly onto Caribou forage quality, especially later in the growing season, though it does show general positive relationships with Caribou forage biomass and digestible energy (Johnson et al. 2018), with greater biomass associated with increased adult survival (Johnson et al. 2022). Expansion in shrub cover has been reported across Arctic tundra ecosystems (Tape et al. 2006; Macander et al. 2022; Nill et al. 2022), which can lead to increases in NDVI (Blok et al. 2011; Fraser et al. 2014; Jespersen et al. 2023). Increases in shrubs alter a wide array of environmental processes such as carbon cycling and snow cover (Myers-Smith et al. 2011; Vowles and Björk 2019; Mekonnen et al. 2021) and have been linked with declines in Caribou populations and key forage species such as lichens (Fraser et al. 2014; Fauchald et al. 2017; Macander et al. 2022). Further investigation is needed into the relationship between NDVI, forage quality, and Caribou habitat use, especially as conditions continue to change in the Arctic. We also note that NDVI was not included in M6a, which was a competing model for fine-scale winter distribution, further reinforcing caution about interpretation of its influence on Caribou winter use patterns.

Insect activity was also positively related to the probability of Caribou migrating south and using the E Brooks wintering area (Figs 4 and 5). Biting insects alter Caribou habitat selection and reduce foraging behavior, calving success, and survival (Russell et al. 1993; Wilson et al. 2012; Joly et al. 2020; Johnson et al. 2022). If these influences lead to reduced body condition, the observation of a greater likelihood to leave the coastal plain for Caribou subjected to more insect activity appears counter to our proposal for NDVI. However, this could suggest compensatory plasticity, with individuals whose foraging is more interrupted by insects being more willing to tolerate the energetic requirements of longer migration to access higher quality winter forage in the Brooks Range (Macander et al. 2022). Recent work with the Western Arctic Herd found that individuals that left the coastal plain to migrate to the Brooks Range and beyond encountered more than 2.5 times as much lichen cover as those that did not (Joly et al. 2025). The hypothesis of migration as compensation for harassment requires additional investigation, but if valid, could be exacerbated by expectations of earlier emergence and increased numbers of biting insects under warming conditions, leading to and extending insect harassment effects (Weladji et al. 2003; Witter et al. 2012; Culler et al. 2015).

Pregnancy status was important at both scales, though there was some overlap in confidence intervals. Nonetheless, spatial patterns were evident in the data. Non-pregnant Caribou were more likely to migrate to the Brooks Range than pregnant individuals (Fig. 4). However, differences were only evident for the eastern wintering areas (Fig. 5). This means pregnant animals were more likely to remain in areas with fewer predators but also less lichen availability. Work with the Mentasta Caribou Herd in Alaska suggested a potential tradeoff during calving of forage abundance for increased safety from predators (Barten et al. 2001). It is possible, based on our findings, that such tradeoffs may also influence winter behavior for the TCH. Unlike some migratory ungulates that “surf the green wave,” migrating in alignment with timing of vegetation phenology to match locations to newly emerged forage (Sawyer and Kauffman 2011; Bischof et al. 2012; Merkle et al. 2016), pregnant Caribou are known to migrate to their calving grounds prior to green-up (Gurarie et al. 2019; Laforge et al. 2021). Remaining on the coastal plain, and especially in the E Coastal wintering area nearest to the calving grounds, may reduce the effects of costly migration on individuals already bearing costs of late-stage gestation and anticipating upcoming costs of lactation, while also reducing predator exposure. Caribou are capital breeders, relying on body stores to fuel lactation for newborn calves (Taillon et al. 2013), so reducing spring movement distances may help retain energetic stockpiles for use after calving.

Alternatively, rather than pregnancy directly affecting risk behavior, it may be indicative of another factor influencing migratory behavior—body condition. Previous work demonstrated a positive relationship between fall body condition and successful pregnancy in Caribou (Cameron et al. 1993). Pregnant animals may thus have a higher body condition and be able to better tolerate winter conditions on the coastal plain, while non-pregnant animals with lower body condition may be more willing to risk predation in the mountains in exchange for greater access to winter forage. This again is a fertile area for investigation with body condition data. Nonetheless, some pregnant TCH Caribou do migrate long distances, as is seen in other herds (Fancy et al. 1989; Cameron et al. 2020). Comparing body condition metrics for individuals that remain in E Coastal over winter with those that migrate to E Brooks or E Coastal, as well as across other neighboring herds, will better clarify interactions between pregnancy, winter distribution, migration, and their fitness outcomes.

### Site fidelity across scales

Our model results affirm that female Caribou of the TCH display some fidelity to wintering areas, reusing areas more than expected under null models (Fig. 3). This result may explain why the TCH have maintained use of the same basic winter areas over decades (Person et al. 2007; Fullman et al. 2021). It also reinforces the importance of comparing observed reuse patterns to null expectations to identify when true fidelity is occurring and when reuse patterns can be explained by other conditions (Picardi et al. 2023). Nonetheless, the TCH do not display obligate fidelity, as multiple wintering areas are used each year (Fig. 2) and individual Caribou show plasticity in their use of wintering areas across years, aligning with our hypothesis and with previous reports of relatively weak winter fidelity for the TCH (Fullman et al. 2021).

Our results also suggest that greater awareness may be warranted to the scale of fidelity. The TCH seems to show relatively high fidelity to their overall wintering areas, using the same general areas consistently over time (Person et al. 2007; Fullman et al. 2021; this study), even as other nearby herds have displayed broader range shifts (Joly et al. 2007, 2010; Gurarie et al. 2024). However, studies have also shown that fidelity may vary across spatial scales for Caribou and other species (Schaefer et al. 2000; Heap et al. 2015; Joly et al. 2021). Our wintering areas are relatively large, so simply examining fidelity as returning to the same wintering area may overlook some of the nuance of use of specific sites within wintering areas. Much as Johnson (1980) defined multiple scales of resource selection, so too there may be a need for additional examination of multiple scales of site fidelity as patterns observed at one scale may not translate to broader or finer scales (Schaefer et al. 2000). How this occurs across species will likely depend on the interplay between perceptive abilities of a species and their use of memory to drive repeated space use (e.g., Cameron et al. 2020).

It is also possible that population size may have an influence on site fidelity patterns. Research considering the 2 largest herds in Alaska has concluded that Caribou show low winter fidelity, shifting wintering ranges over time (Morrison et al. 2021; Gurarie et al. 2024). It has even been suggested that shifts in range use are a key adaptation strategy for Caribou (Gurarie et al. 2024). This pattern contrasts, however, with the consistency in wintering area use shown by the smaller TCH over a 30 yr period (Person et al. 2007; Fullman et al. 2021; this study). Research across taxa has found that population size can influence fidelity patterns. Site fidelity decreased as colony size increased for cliff swallows (*Petrochelidon pyrrhonota*), likely related to changing ectoparasite levels (Brown et al. 2017). Bison (*Bison bison*) in Canada showed greater site fidelity as the population decreased (Merkle et al. 2015). However, site fidelity and population density were positively correlated in Brown Trout (*Salmo trutta*; Slavík and Horký 2019). The extent to which population size influences fidelity in Caribou is unknown, though an inverse relationship between population size and site fidelity has been suggested (Schaefer and Mahoney 2013) and large herds show less interchange with adjacent herds compared to smaller herds (Prichard et al. 2020b), with some smaller herds more sedentary than larger herds (Bergerud 1988; Valkenburg et al. 2016). Early work proposed “centers of habitation” for Alaskan Caribou, which are core ranges consistently used at times of both low and high population size (Skoog 1968). High fidelity would be expected for such areas. As Caribou populations grow they may expand beyond their center of habitation, contracting again as the population declines (Skoog 1968). The Western Arctic Herd appears to have maintained plasticity in its winter distribution despite having declined by nearly 70% from its peak in the early 2000s (Gurarie et al. 2024). Nonetheless, its current size is still about 2.5 times larger than that of the TCH, encouraging further investigation into the interplay between population size and site fidelity in Caribou.

We also note that all collared Caribou in this study were females. Males may show different patterns of behavioral plasticity and fidelity as differences in body size, pregnancy, energetic requirements and other factors lead to differential risks and rewards across sexes (Chauveau et al. 2025). Sex differences in site fidelity have been recorded in a variety of species (Sinsch 1992; Craig and Herman 1997; Sztukowski et al. 2018) but are not ubiquitous (Cameron et al. 2007; Rueger et al. 2014). This is an area that warrants further investigation.

### Recent changes in winter distribution

Unlike other Alaskan Caribou herds that have exhibited highly variable wintering area behavior (e.g., Western Arctic Herd; Joly et al. 2007, 2010; Gurarie et al. 2024), the TCH has shown remarkable consistency in its winter distribution over time (Person et al. 2007; Fullman et al. 2021). Our results continue to affirm use of the 4 general wintering areas previously identified (Person et al. 2007; Fullman et al. 2021). Nonetheless, we noted shifts in proportional use of wintering areas between 2004 to 2015 and 2016 to 2020, with increased use of E Coastal and E Brooks in the latter period combined with a lack of use of W Brooks by collared Caribou. This pattern may reflect broader trends in the Alaskan Arctic as the Central Arctic Herd to the east of the TCH also demonstrated a shift in winter distribution after the 2015 analysis year, with more individuals wintering north of the continental divide (Pedersen et al. 2021). The Western Arctic Herd to the southwest of the TCH also exhibited changes in winter distribution in recent years, including shifts in primary winter range and increased prevalence of nonmigratory individuals (Gurarie et al. 2024; Joly et al. 2025). The breakpoint for these shifts was identified as analogous to our 2016 analysis-year (Gurarie et al. 2024). Similarly, the Western Arctic Herd largely abandoned its southwestern winter range on the Seward Peninsula around the same time the TCH showed no use of the W Brooks wintering area (Gurarie et al. 2024). It is striking that all 3 of these herds demonstrated shifts in behavior in a similar timeframe. Synchrony has been noted for Caribou behavior during spring migration (Gurarie et al. 2019), but further investigation is needed to investigate this apparent synchrony of winter behavior and drivers of these patterns, as well as continued monitoring to reveal whether they persist, which will help inform whether changing winter behavior indicates alteration of winter fidelity patterns or just temporary deviations from historic usage.

If winter distribution patterns continue to shift for Alaskan Caribou, it could have implications for human–Caribou interactions. The E Coastal wintering area that has seen increased use by the TCH in recent years overlaps with areas of existing oil and gas leases and infrastructure. The sensitivity of calving Caribou to infrastructure and human activity has been well documented (Dau and Cameron 1986; Cameron et al. 1992, 2005; Johnson et al. 2020; Prichard et al. 2020a), as have reactions at other seasons of the year, such as during migration (Wilson et al. 2016; Johnson et al. 2020; Severson et al. 2023; Boulanger et al. 2024; Fullman et al. 2025), but less information is available about how Caribou respond to infrastructure and activity during the winter. Increased stress hormones and altered distribution and movement behavior have been documented around seasonal petroleum infrastructure in Canada (Smith and Johnson 2023; Smith et al. 2023), underscoring a need for continued monitoring of interactions of the TCH and other Caribou herds with human activity and infrastructure to inform management approaches.

In conclusion, improved understanding of prevalence, extent, and mechanisms of migration and its changing patterns is essential to inform effective management (Xu et al. 2021). While at a herd scale TCH fidelity appears high to historically used wintering areas, at finer scales, individuals show some fidelity but a fairly high degree of plasticity in winter distribution from year to year. We identify multiple intrinsic and extrinsic factors that appear associated with TCH winter behavior and suggest possible mechanisms for future investigation. There are, however, additional factors we were not able to analyze, such as body condition and disease dynamics, that also warrant future study. While behavioral plasticity can convey resilience to environmental change (Xu et al. 2021; Merkle et al. 2022), this is only possible if species are able to respond quickly enough to adapt to rapidly shifting conditions. The Arctic is warming faster than the global average, with many resulting and anticipated ecological changes (Post et al. 2019). It is crucial to continue to monitor movement, behavioral plasticity, and site fidelity for large herbivores and other species to identify how they are responding to changing conditions. Linking observed changes in migration and winter distribution patterns with fitness outcomes like survival and reproduction (e.g., Gurarie et al. 2024) will also play a key role in understanding how such changes may affect populations.

## Supplementary Material

gyag039_Supplementary_Data

## Data Availability

Derived data used in our analyses are available via the Dryad repository (Fullman et al. 2026).

## Supplementary data

**Supplementary Data SD1**. Environmental covariates for the 4 wintering areas used by the Teshekpuk Caribou Herd.

**Supplementary Data SD2**. Annual winter utilization distributions for Teshekpuk Caribou Herd females in northern Alaska, analysis-years 2004 to 2020.

**Supplementary Data SD3**. Population-level winter utilization distribution for Teshekpuk Caribou Herd females in northern Alaska.

**Supplementary Data SD4**. Overlap statistics between 2004 to 2015 and 2004 to 2020 winter contours.

**Supplementary Data SD5**. Spatial overlap between 2004 to 2015 and 2004 to 2020 winter contours.

**Supplementary Data SD6**. Sample sizes for wintering area use by Teshekpuk Caribou Herd females in northern Alaska, 2004 to 2020.

**Supplementary Data SD7**. Observed and simulated wintering area transition probabilities.

**Supplementary Data SD8**. Multinomial model expectations under each null model for Teshekpuk Caribou Herd female wintering area use, 2004 to 2020.

**Supplementary Data SD9**. Model validation results for the probability of Teshekpuk Caribou Herd females leaving the Alaskan coastal plain overwinter.

**Supplementary Data SD10**. Model summary for the top-ranked model explaining likelihood of Teshekpuk Caribou Herd females leaving the Alaskan coastal plain overwinter.

**Supplementary Data SD11**. Partial effect plots for the probability of Teshekpuk Caribou Herd females leaving the coastal plain overwinter.

**Supplementary Data SD12**. Model validation results for the probability of Teshekpuk Caribou Herd females using specific wintering areas.

**Supplementary Data SD13**. Model summary for competing models explaining likelihood of Teshekpuk Caribou Herd females using specific wintering areas.

**Supplementary Data SD14**. Partial effect plots for the probability of overwintering in W Coastal and E Brooks by Teshekpuk Caribou Herd females.
