# Supplementary material for: Caribou balance winter range fidelity and plasticity in response to weather, pregnancy, and summer range conditions
Source: J Mammal. 2026 May 23;107(4):696–713. doi: 10.1093/jmammal/gyag039 (PMC13416195; doi:10.1093/jmammal/gyag039)
Supplement: gyag039_Supplementary_Data [file gyag039_supplementary_data.zip › Fullman et al Supplementary Data 2025-12-11.pdf]

Fullman TJ,[1] Person BT, Karpovich S, Prichard AK, Hepler J, Zuur AF. Caribou balance winter range fidelity and plasticity in response to weather, pregnancy, and summer range conditions. Journal of Mammalogy.

1. Corresponding author: The Wilderness Society, Anchorage, AK, USA.
tim_fullman@tws.org

Supplementary Data:
Additional Tables and Figures

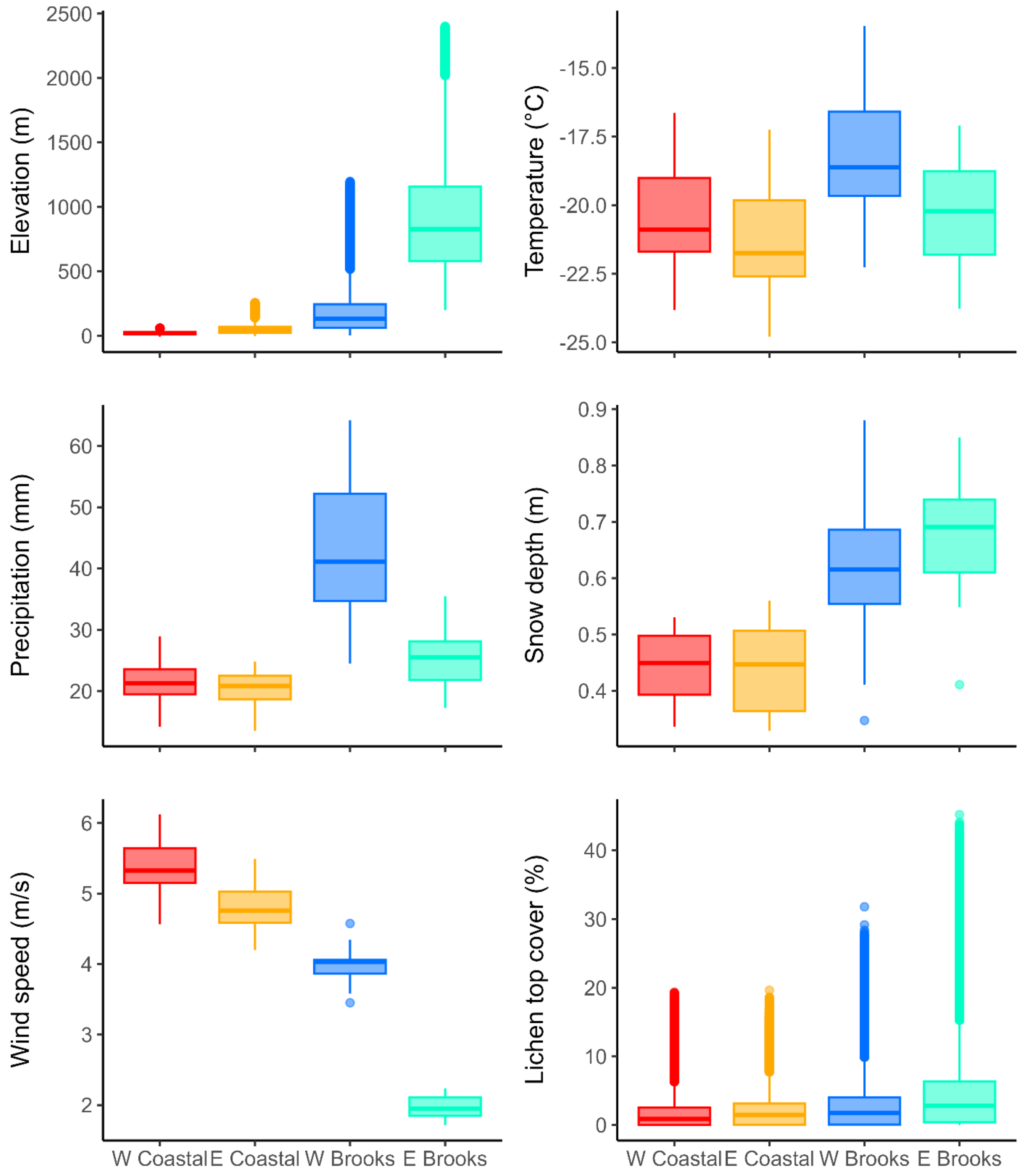

Supplementary Data SD1. Environmental covariates for the four wintering areas used by the Teshekpuk Caribou Herd in northern Alaska, analysis-years 2004-2020. Elevation data represent a summary of all 32 m elevation pixels from the ArcticDEM mosaic dataset (Porter *et al.* 2023). Weather data represent winter season (11 Nov – 1 May) mean values for each analysis-year, obtained from hourly ERA5-Land reanalysis data (Muñoz-Sabater *et al.* 2021) accessed from Google Earth Engine (Gorelick *et al.* 2017; Muñoz-Sabater 2019). Lichen data summarize 30 m pixels from modeled lichen top cover for 2020 (Macander and Nelson 2022; Macander *et al.* 2022).

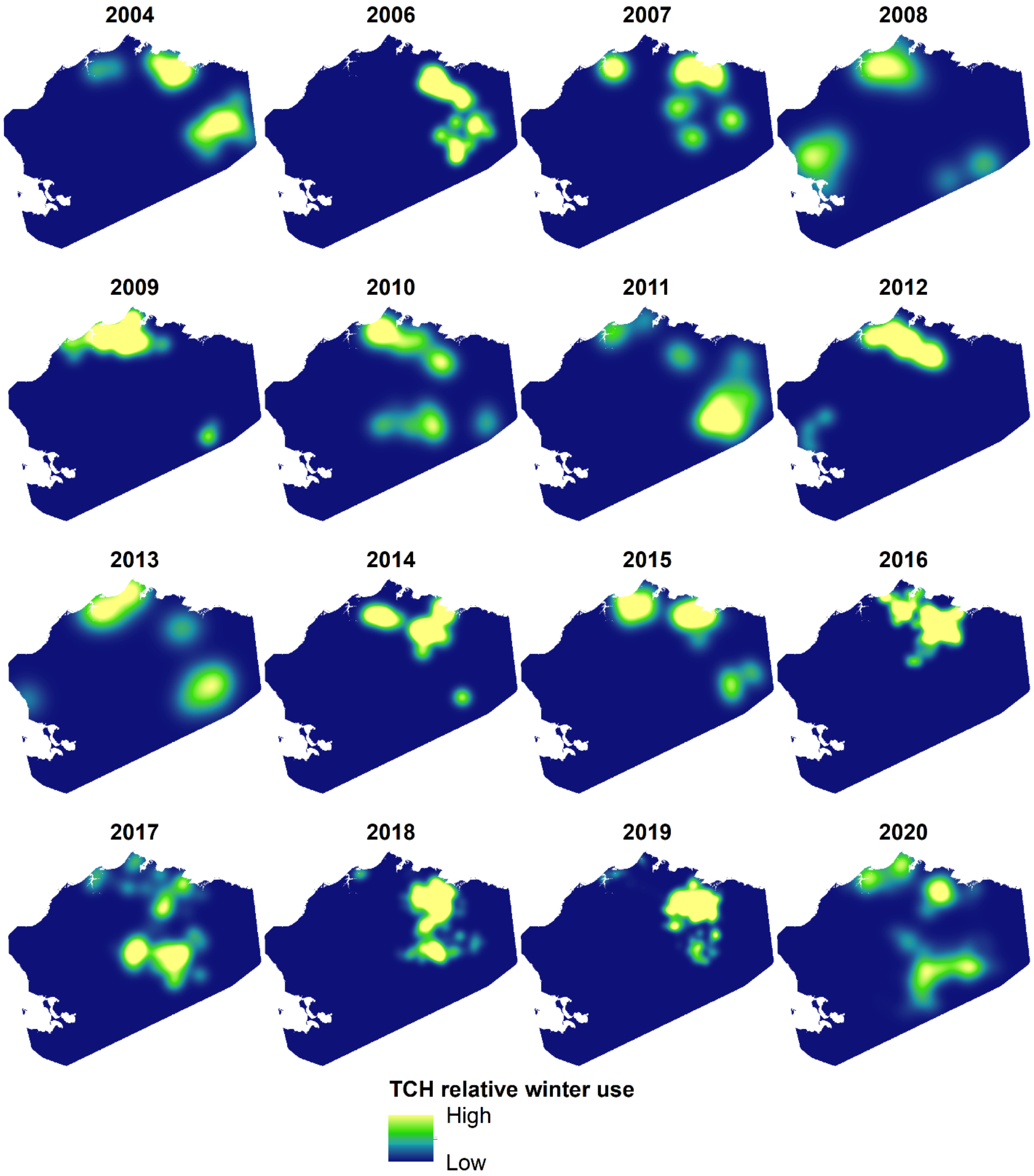

Supplementary Data SD2. Annual winter utilization distributions for Teshekpuk Caribou Herd females in northern Alaska, analysis-years 2004–2020. Winter use distributions are standardized to all sum to one. Years represent analysis-years spanning from 1 Jul of the indicated year to 30 Jun of the next year.

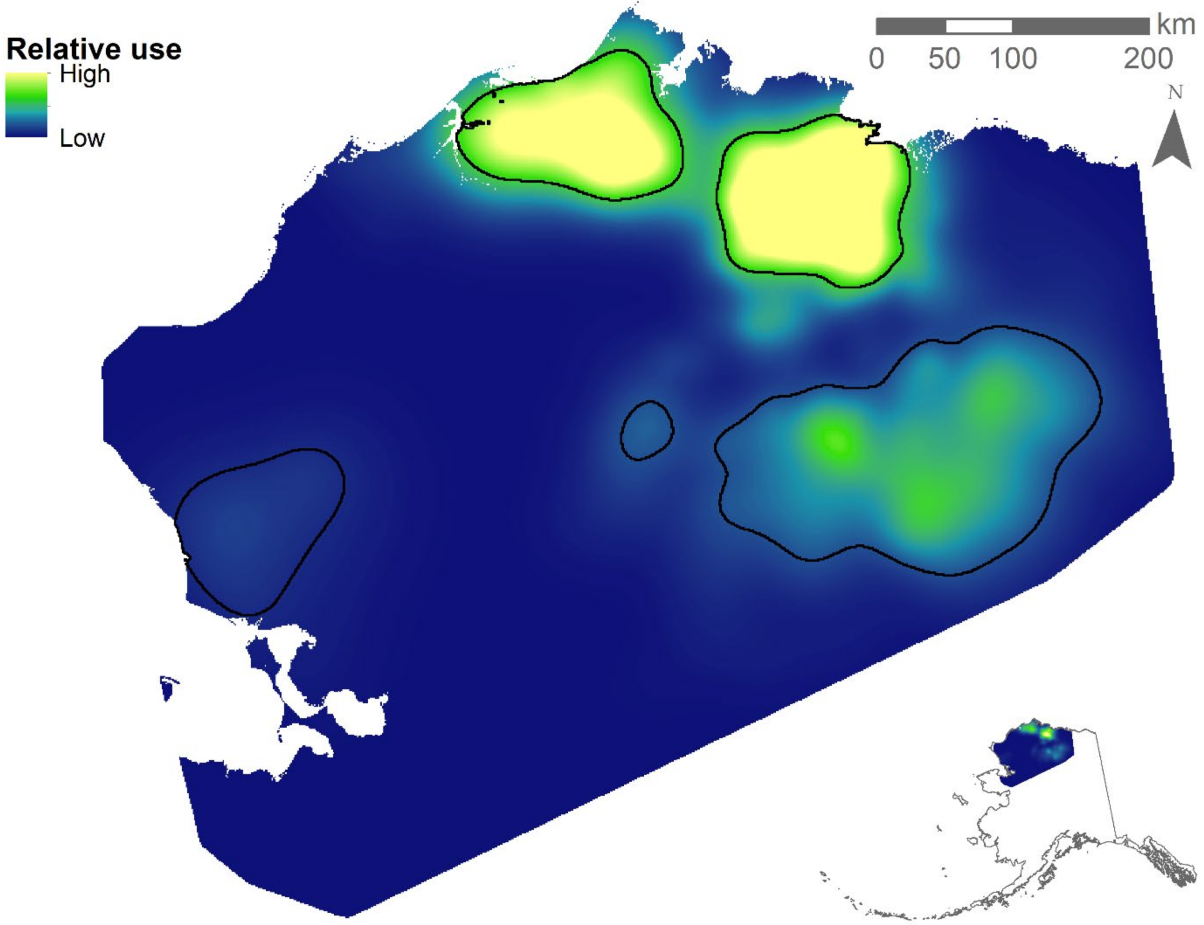

Supplementary Data SD3. Population-level winter utilization distribution for Teshekpuk Caribou Herd females in northern Alaska, analysis-years 2004–2020. Winter area contours (black lines) were derived from the population-level winter data.

Supplementary Data SD4. Overlap statistics between the 2004–2020 winter contours analyzed in this study and those spanning 2004–2015 reported by Fullman et al. (2021). Area of each wintering contour and overlap between the two periods (km^2) are reported, along with the percent coverage of the 2004–2015 contours by the 2004–2020 contours from this study. Note that all data used to identify the 2004–2015 contours also were included in the 2004–2020 contours so differences reflected the addition of data from the 2016–2020 analysis-years.

	W Coastal	E Coastal	W Brooks	E Brooks
Winter area 2004–2015 (km^2)	15,070	10,043	7,279	28,852
Winter area 2004–2020 (km^2)	12,423	14,234	10,027	36,218
Area of overlap (km^2)	12,423	10,038	6,975	26,168
% coverage of 2004–2015 contours by 2004–2020 contours	82.4%	100.0%	95.8%	90.7%

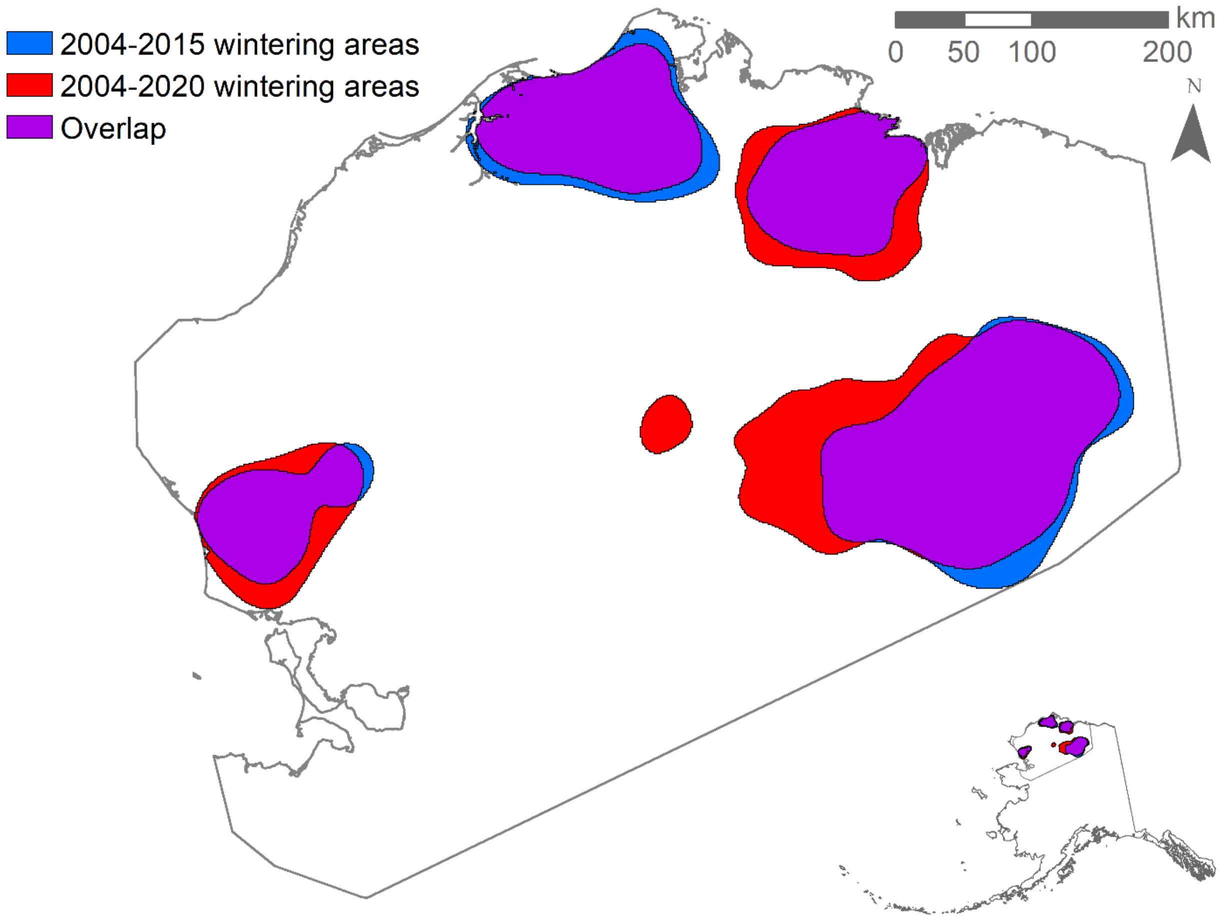

Supplementary Data SD5. Spatial overlap between the 2004–2020 winter contours analyzed in this study and those spanning 2004–2015 reported by Fullman et al. (2021). See Supplementary Data S4 for overlap statistics. Note that all data used to identify the 2004–2015 contours also were included in the 2004–2020 contours so differences reflected the addition of data from the 2016–2020 analysis-years.

Supplementary Data SD6. Sample sizes for wintering area use by Teshekpuk Caribou Herd females in northern Alaska, 2004–2020. Records analyzed in this study spanned the full time period. Data from 2004–2015 match results of Fullman et al. (2021), which were identical to classifications in this study using updated winter use area contours. Records from 2016–2020 were added in this study. All records were analyzed jointly in this paper. Dates reflect analysis-years, indicating a time period from 1 Jul of the indicated year until 30 Jun of the following year (e.g., the 2016 analysis-year spans 1 Jul 2016 – 30 Jun 2017).

Analysis-year	W Coastal	E Coastal	W Brooks	E Brooks
2004–2015	71	43	10	40
2016–2020	51	169	0	124
2004–2020	122	212	10	164

Supplementary Data SD7. Observed and simulated transition probabilities (mean and 95% confidence interval) for all wintering area transitions of Teshekpuk Caribou Herd females in northern Alaska, 2004–2020, under four null hypotheses. Cst = Coastal, Brks = Brooks, equal = equal probability of use, area = area-weighted probability of use, distance = distance-weighted probability of use, pop = population-weighted probability of use.

			**equal**			**area**			**distance**			**pop**		
Year 1	Year 2	obs	mean	CI_{low}	CI_{up}	mean	CI_{low}	CI_{up}	mean	CI_{low}	CI_{up}	mean	CI_{low}	CI_{up}
W Cst	W Cst	0.55	0.25	0.25	0.26	0.17	0.16	0.17	0.29	0.29	0.29	0.24	0.24	0.25
W Cst	E Cst	0.24	0.25	0.25	0.26	0.20	0.19	0.20	0.31	0.30	0.31	0.42	0.41	0.42
W Cst	W Brks	0.00	0.25	0.24	0.25	0.14	0.13	0.14	0.16	0.16	0.17	0.02	0.02	0.02
W Cst	E Brks	0.21	0.25	0.24	0.25	0.50	0.49	0.50	0.24	0.23	0.24	0.32	0.32	0.33
E Cst	W Cst	0.17	0.25	0.25	0.26	0.17	0.17	0.18	0.29	0.28	0.29	0.24	0.24	0.24
E Cst	E Cst	0.48	0.25	0.24	0.25	0.20	0.19	0.20	0.31	0.30	0.31	0.41	0.41	0.42
E Cst	W Brks	0.00	0.25	0.24	0.25	0.14	0.13	0.14	0.16	0.16	0.17	0.02	0.02	0.02
E Cst	E Brks	0.34	0.25	0.25	0.26	0.49	0.49	0.50	0.24	0.24	0.24	0.33	0.32	0.33
W Brks	W Cst	0.88	0.25	0.25	0.26	0.17	0.16	0.17	0.29	0.28	0.29	0.24	0.22	0.25
W Brks	E Cst	0.13	0.25	0.25	0.26	0.20	0.19	0.20	0.31	0.31	0.32	0.43	0.41	0.45
W Brks	W Brks	0.00	0.25	0.24	0.25	0.14	0.13	0.14	0.16	0.16	0.17	0.02	0.01	0.02
W Brks	E Brks	0.00	0.25	0.24	0.25	0.50	0.49	0.50	0.24	0.23	0.24	0.31	0.29	0.33
E Brks	W Cst	0.08	0.25	0.25	0.26	0.17	0.17	0.18	0.29	0.28	0.29	0.24	0.24	0.25
E Brks	E Cst	0.49	0.25	0.25	0.26	0.19	0.19	0.20	0.31	0.30	0.31	0.42	0.41	0.42
E Brks	W Brks	0.02	0.25	0.24	0.25	0.14	0.13	0.14	0.17	0.16	0.17	0.02	0.02	0.02
E Brks	E Brks	0.40	0.25	0.25	0.25	0.49	0.49	0.50	0.23	0.23	0.24	0.32	0.31	0.32

Supplementary Data SD8. Multinomial model expectations under each null model for Teshekpuk Caribou Herd female wintering area use, 2004–2020. Values indicate the probability of observing the recorded distribution of wintering area use in every analysis-year with data under the given null hypothesis. Hypothesis definitions match Supplementary Data S7. Sample size per analysis-year (n) only includes caribou with multiple years of data that were used in calculation of transition probabilities.

analysis-year	n	equal	area	distance	pop
2004	5	0.029	0.048	0.046	0.131
2006	3	0.047	0.145	0.052	0.130
2007	8	0.003	0.002	0.008	0.053
2008	20	<0.001	<0.001	<0.001	<0.001
2009	18	<0.001	<0.001	<0.001	<0.001
2010	12	<0.001	0.003	0.002	0.008
2011	9	<0.001	0.007	<0.001	<0.001
2012	10	<0.001	<0.001	<0.001	<0.001
2013	15	<0.001	<0.001	<0.001	<0.001
2014	16	<0.001	<0.001	<0.001	0.002
2015	17	<0.001	<0.001	<0.001	0.004
2016	40	<0.001	<0.001	<0.001	<0.001
2017	56	<0.001	<0.001	<0.001	<0.001
2018	61	<0.001	<0.001	<0.001	<0.001
2019	81	<0.001	<0.001	<0.001	<0.001
2020	73	<0.001	<0.001	<0.001	<0.001
all	444	<0.001	<0.001	<0.001	<0.001

Supplementary Data SD9. Model validation results for the coarse-scale analysis of the probability of Teshekpuk Caribou Herd females migrating south to the Brooks Range mountains overwinter, 2004–2020. ΔAIC = difference between the current model and model with the lowest Akaike's Information Criterion, AUC = area under the receiver operating characteristic curve, PCC = percent correctly classified, DevExp = deviance explained. See Table 1 for model definitions.

Rank	Model	ΔAIC	AUC	LogLoss	PCC	Sensitivity	Specificity	DevExp
1	M7	0.00	0.88	0.40	83.20	0.66	0.91	58.24
2	M6a	10.26	0.89	0.39	81.17	0.64	0.89	54.76
3	M6b	23.25	0.87	0.41	82.17	0.64	0.91	54.41
4	M9	99.75	0.78	0.53	74.55	0.52	0.86	43.54
5	M8	118.33	0.76	0.55	69.22	0.41	0.84	39.09
6	M5	150.09	0.69	0.60	70.47	0.33	0.89	31.99
7	M1	162.75	0.65	0.62	67.45	0.24	0.89	25.77
8	M3	168.91	0.66	0.61	68.46	0.20	0.92	25.22
9	M4	172.22	0.66	0.62	66.67	0.26	0.88	25.29
10	M6c	176.58	0.64	0.62	66.15	0.16	0.91	21.85
11	M10	188.99	0.59	0.63	64.64	0.02	0.95	17.02
12	M2	192.27	0.56	0.64	64.39	0.04	0.93	16.91

Supplementary Data SD10. Model summary for M7, the top-ranked model explaining likelihood of Teshekpuk Caribou Herd females migrating south overwinter, 2004–2020. s() indicates a regression spline for the indicated variable. Lower case letters preceding covariates indicate seasonal values (s = summer, f = fall). NDVI = normalized difference vegetation index, insect = insect harassment index, Temp = air temperature, Precip = precipitation, edf = effective degrees of freedom, SE = standard error.

**Parametric Parameters**	**Estimate**	**SE**
Intercept	-0.797	0.273
Pregnant	-0.726	0.330
**Smoothed Parameter**	**edf**	**Chi-square**
s(NDVI)	0.905	9.870
s(insect)	0.890	7.470
s(age) × non-pregnant	<0.001	0.000
s(age) × pregnant	<0.001	0.000
s(sTemp)	1.707	9.001
s(sPrecip)	0.523	1.017
s(fTemp)	1.766	69.126
s(fPrecip)	0.939	14.966
s(caribou)	32.530	41.789

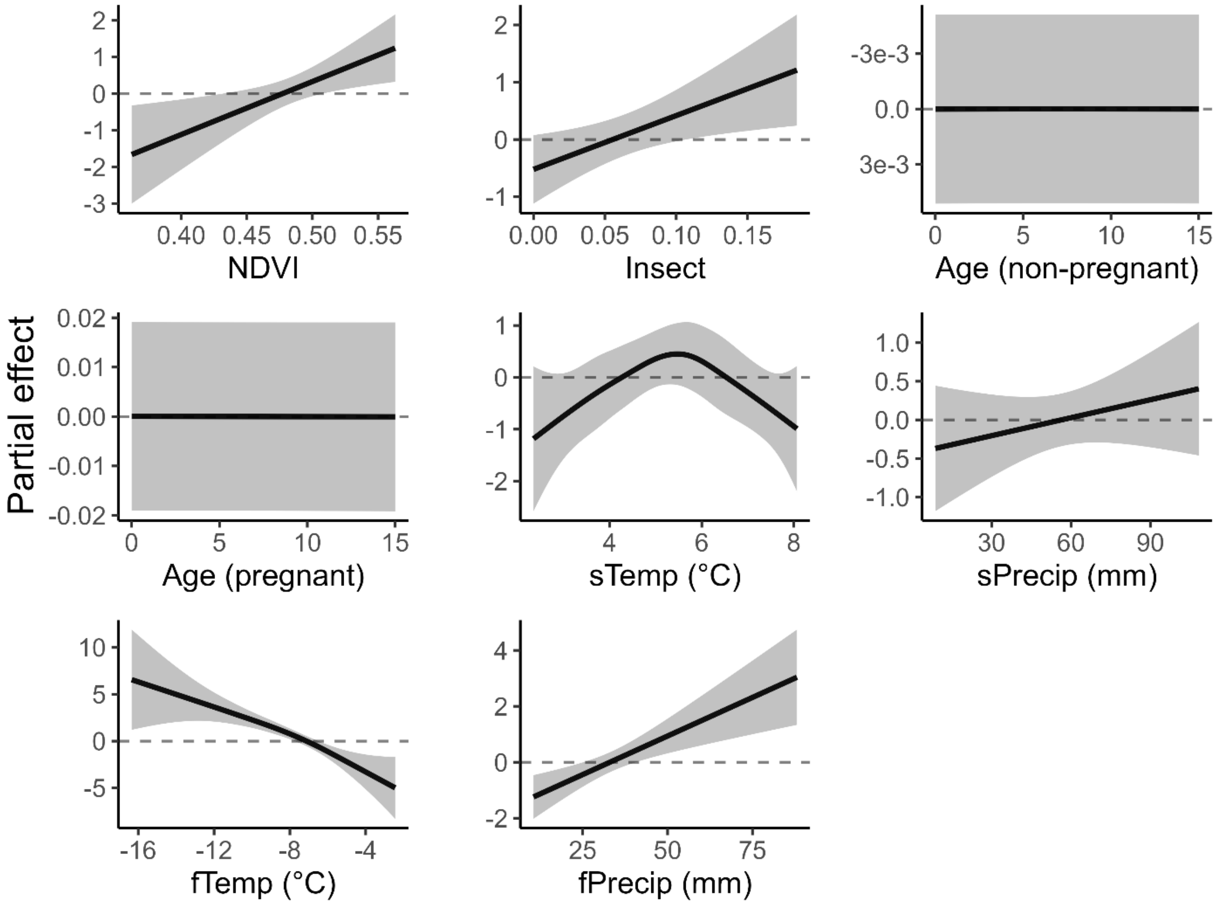

Supplementary Data SD11. Partial effect plots depicting smoothers with simultaneous 95% confidence intervals under M7 for the probability of Teshekpuk Caribou Herd females migrating south overwinter in northern Alaska, 2004–2020. Lower case letters preceding covariates indicate seasonal values (s = summer, f = fall). NDVI = normalized difference vegetation index, Insect = insect harassment index, Temp = air temperature, Precip = precipitation.

Supplementary Data SD12. Model validation results for fine-scale analysis of the probability of Teshekpuk Caribou Herd females using specific wintering areas, 2004–2021. ΔAIC = difference between the current model and model with the lowest Akaike's Information Criterion, AUC = area under the receiver operating characteristic curve, PCC = percent correctly classified, DevExp = deviance explained. See Table 1 for model definitions.

**Rank**	**Model**	**ΔAIC**	**AUC**	**LogLoss**	**PCC**	**Sensitivity**	**Specificity**	**DevExp**
1	M7	0.00	0.86	0.72	69.00	0.69	0.88	54.90
2	M6a	0.69	0.85	0.72	69.27	0.69	0.89	49.24
3	M8	58.72	0.80	0.88	63.80	0.64	0.86	52.21
4	M9	68.20	0.81	0.86	61.99	0.62	0.85	55.54
5	M6b	69.22	0.79	0.84	63.27	0.63	0.86	46.44
6	M5	142.32	0.73	0.95	55.23	0.55	0.82	38.16
7	M1	167.44	0.74	0.92	55.20	0.55	0.82	31.56
8	M6c	178.67	0.69	0.99	48.69	0.49	0.79	30.55
9	M4	211.25	0.67	1.01	48.17	0.48	0.79	28.89
10	M3	219.42	0.62	1.08	46.86	0.47	0.78	28.86
11	M2	233.05	0.67	1.02	47.66	0.48	0.79	23.44
12	M10	236.79	0.64	1.04	46.09	0.46	0.78	23.91

Supplementary Data SD13. Model summary for competing models explaining likelihood of Teshekpuk Caribou Herd females using specific wintering areas, 2004–2020. See Table 1 for model definitions. The Area column indicates whether results report modifications of the reference model (E Coastal) for W Coastal (WC) or E Brooks (EB). Due to small sample size W Brooks was not included in this analysis. Other terms and abbreviations follow Supplementary Data S10.

		M7		M6a	
**Area**	**Parametric Parameter**	**Estimate**	**SE**	**Estimate**	**SE**
WC	Intercept	-1.095	0.376	-1.129	0.216
WC	Pregnant	-0.184	0.407		
EB	Intercept	-0.514	0.312	-0.989	0.223
EB	Pregnant	-0.771	0.364		
**Area**	**Smoother Parameter**	**edf**	**Chi-square**	**edf**	**Chi-square**
WC	s(NDVI)	2.074	15.121		
WC	s(insect)	0.061	0.061		
WC	s(age) × non-pregnant	3.255	20.947		
WC	s(age) × pregnant	0.521	1.087		
WC	s(sTemp)	0.814	3.762	0.955	19.487
WC	s(sPrecip)	3.094	16.781	3.013	16.781
WC	s(fTemp)	1.631	16.295	1.790	16.901
WC	s(fPrecip)	0.285	0.340	<0.001	0.000
WC	s(caribou)	21.150	25.918	26.510	34.222
EB	s(NDVI)	0.696	2.034		
EB	s(insect)	0.881	6.746		
EB	s(age) × non-pregnant	0.840	2.082		
EB	s(age) × pregnant	<0.001	0.000		
EB	s(sTemp)	2.074	12.694	3.004	21.734
EB	s(sPrecip)	0.696	1.981	0.900	8.234
EB	s(fTemp)	2.207	46.996	2.898	46.179
EB	s(fPrecip)	0.949	17.941	0.946	16.883
EB	s(caribou)	23.720	28.444	19.640	23.087

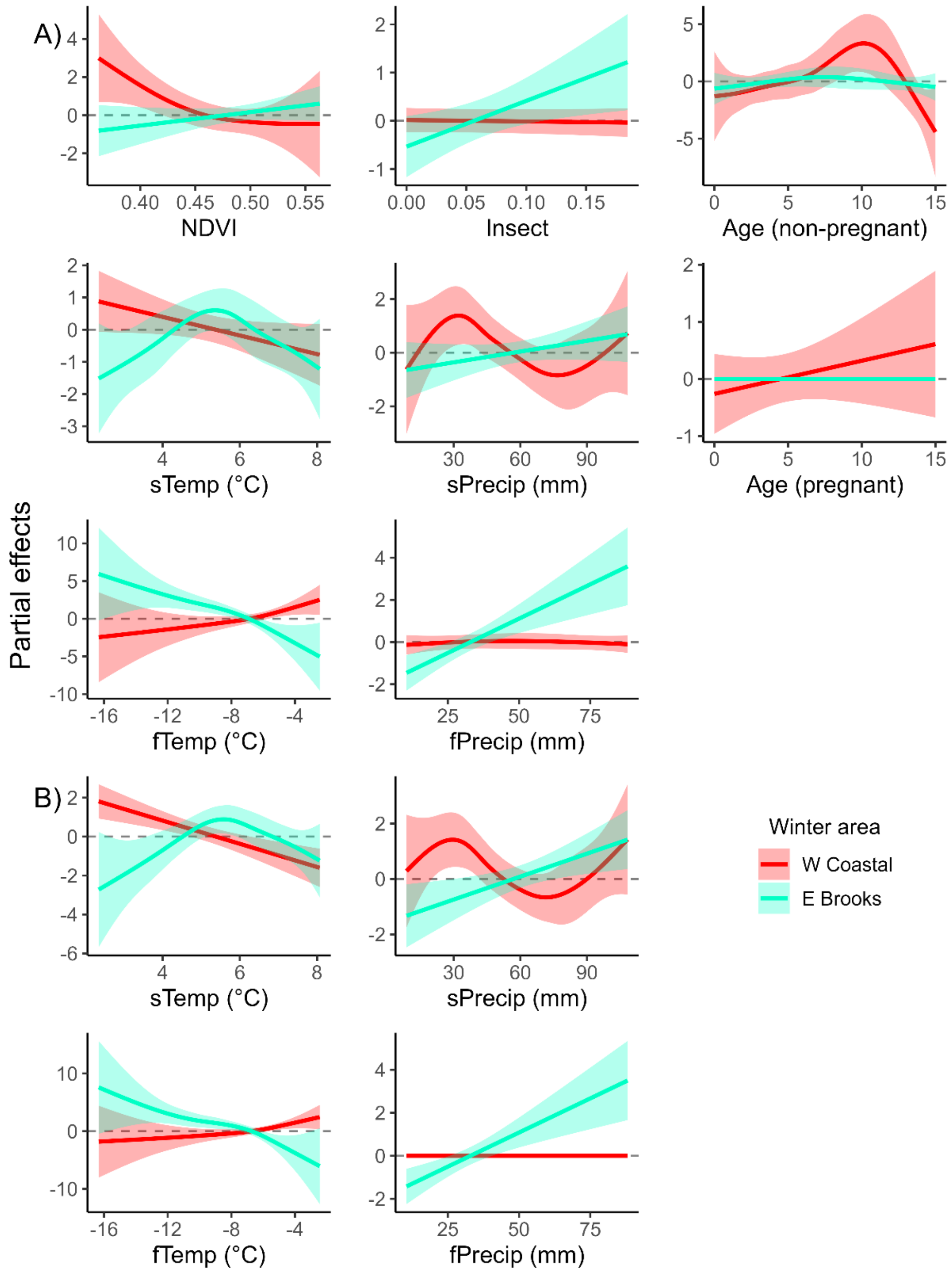

Supplementary Data SD14. Partial effect plots depicting smoothers with simultaneous 95% confidence intervals under a) M7 and b) M6a for the probability of overwintering in W Coastal (red) and E Brooks (turquoise) by Teshekpuk Caribou Herd females in northern Alaska, 2004–2020. Models were run using E Coastal as the base condition. W Brooks was not included due to small sample size. Lower case letters preceding covariates indicate seasonal values (s = summer, f = fall). NDVI = normalized difference vegetation index, Insect = insect harassment index, Temp = air temperature, Precip = precipitation.

References

Fullman TJ, Person BT, Prichard AK, Parrett LS. 2021. Variation in winter site fidelity within and among individuals influences movement behavior in a partially migratory ungulate. PLOS ONE 16(9):e0258128. https://doi.org/10.1371/journal.pone.0258128

Gorelick N, Hancher M, Dixon M, Ilyushchenko S, Thau D, Moore R. 2017. Google Earth Engine: Planetary-scale geospatial analysis for everyone. Remote Sensing of Environment 202:18–27. https://doi.org/10.1016/j.rse.2017.06.031

Macander MJ, Nelson PR. 2022. Arctic-Boreal Vulnerability Experiment (ABoVE): Modeled Top Cover by Plant Functional Type over Alaska and Yukon, 1985-2020. https://doi.org/10.3334/ORNLDAAC/2032.

Macander MJ, Nelson PR, Nawrocki TW, Frost GV, Orndahl KM, Palm EC, Wells AF, Goetz SJ. 2022. Time-series maps reveal widespread change in plant functional type cover across Arctic and boreal Alaska and Yukon. Environmental Research Letters 17(5):054042. https://doi.org/10.1088/1748-9326/ac6965

Muñoz-Sabater J. 2019. ERA5-Land monthly averaged data from 1981 to present. Copernicus Climate Change Service (C3S) Climate Data Store (CDS). https://developers.google.com/earth-engine/datasets/catalog/ECMWF_ERA5_LAND_HOURLY.

Muñoz-Sabater J, Dutra E, Agustí-Panareda A, Albergel C, Arduini G, Balsamo G, Boussetta S, Choulga M, Harrigan S, Hersbach H *et al.* 2021. ERA5-Land: a state-of-the-art global reanalysis dataset for land applications. Earth System Science Data 13(9):4349–4383. https://doi.org/10.5194/essd-13-4349-2021

Porter C, Howat I, Noh MJ, Husby E, Khuvis S, Danish E, Tomko K, Gardiner J, Negrete A, Yadav B *et al.* 2023. ArcticDEM - Mosaics. Version 4.1. Harvard Dataverse, V1. [accessed 25 Mar 2025]. https://doi.org/10.7910/DVN/3VDC4W.
